# Adapting a South African social innovation for maternal peer support to migrant communities in Sweden: a qualitative study

**DOI:** 10.1186/s12939-022-01687-4

**Published:** 2022-06-22

**Authors:** Per Kåks, Anna Bergström, Sibylle Herzig van Wees, Mats Målqvist

**Affiliations:** 1 SWEDESD, Department of Women’s and Children’s Health, Uppsala University, 75236 Uppsala, Sweden; 2 Department of Global Public Health, Karolinska Institute, 171 76 Stockholm, Sweden

**Keywords:** Health inequity, Paraprofessionals, Home visiting, Social determinants of health, Migration, Segregation, Social integration, Early childhood development, Social innovation

## Abstract

**Introduction and aim:**

Social and health disparities persist in Sweden despite a high quality and universally accessible welfare system. One way of bridging social gaps is through social innovations targeting the most vulnerable groups. The South African Philani model, a social innovation for peer support aimed at pregnant women and mothers of young children, was adapted to the local context in southern Sweden. This study aimed to document and analyze the process of adapting the Philani model to the Swedish context.

**Methods:**

Eight semi-structured interviews and three workshops were held with eleven stakeholders and peer supporters in the implementing organization and its steering committee. The data were analyzed using thematic analysis.

**Results:**

The analysis resulted in five main themes and fifteen sub-themes representing different aspects of how the peer support model was contextualized. The main themes described rationalizations for focusing on social determinants rather than health behaviors, using indirect mechanisms and social ripple effects to achieve change, focusing on referring clients to established public and civil society services, responding to a heterogeneous sociocultural context by recruiting peer supporters with diverse competencies, and having a high degree of flexibility in how contact was made with clients and how their needs were met.

**Conclusion:**

The South African Philani model was contextualized to support socially disadvantaged mothers and expectant mothers among migrant communities in Sweden. In the process, adaptations of the intervention’s overall focus, working methods, and recruitment and outreach strategies were motivated by the existing range of services, the composition of the target group and the conditions of the delivering organization. This study highlights various considerations that arise when a social innovation developed in a low- or middle-income context is implemented in a high-income context.

**Supplementary Information:**

The online version contains supplementary material available at 10.1186/s12939-022-01687-4.

## Introduction

Children need stimulating and nurturing conditions if they are to reach their full potential. This includes access to early childhood education and adequate material standards in the home [1, 2]. Previous research has shown that children growing up in less supportive and stimulating environments are at a greater risk of not developing good social, emotional and practical skills than those growing up in more stimulating environments [3]. Suboptimal conditions in early life can have long-lasting effects on wellbeing and opportunities later in life [4]. Parents’ resources and engagement in their children are thus essential for creating health and wellbeing opportunities throughout life. Maternal health during pregnancy and the first year of the child's life is also important for the child’s cognitive and social development [5], displaying how parents’ health is intertwined with their children’s.

Despite a well-developed and accessible welfare system in Sweden, inequalities in health persist. Non-Western immigrants are overrepresented on the lower end of the socioeconomic spectrum, and as a consequence, children in immigrant-dense areas experience worse health than those in areas dominated by the ethnic majority [6]. Specifically, these differences include disparities in outcomes such as obesity, dental health and exposure to tobacco smoke [7–9]. A social gradient can also be seen for utilization of welfare services in Sweden, in the form of lower participation in early childhood education and higher consumption of emergency care among disadvantaged groups [10, 11].

### Social innovations for empowerment

Persisting social and health disparities must be met with innovative solutions [12]. Such solutions can entail social innovations, which has been defined by Mulgan and Pulford [13] as “new ideas (products, services and models) that simultaneously meet social needs (more effectively than alternatives) and create new social relationships or collaborations.” Third sector organizations can take on roles of both service providers and advocates for disadvantaged groups, using social innovations to both complement and compensate for shortcomings in rigid public systems [14]. However, their alternative roles to the public sector may also be limited by factors such as conditional public funding [15].

Social innovations that have proven to work on a small scale, in one context, can be scaled to increase their impact. This process is dependent on several conditions, including ‘pull’ factors (i.e. demand in the form of societal need coupled with financial capacity), ‘push’ factors (i.e. supply in the form of a feasible and effective innovation), strategies that connect supply and demand (e.g. organizational forms), and adaptation to the environment in which it is scaled [13]. However, in many cases, social innovations targeted at disadvantaged groups rely on external funding. With this in mind, it is often not relevant to apply a market model of demand, but rather to see the demand for social innovations as an interplay between the interests of different actors such as recipients and financers [16].

One type of service-related social innovation is peer support, which strives to increase social support capital within groups with certain characteristics. Peer support for maternal and child health comes in many forms, ranging from practically focused community health worker services to purely emotional support [17]. Some peer support models are rooted in an idea of promoting empowerment, i.e., generating the attitudinal change needed to increase agency in managing one’s life and claiming individual rights. The concept of peer support for empowerment has been described by Brodsky and Cattaneo [18] as a process that is experienced internally and enacted socially, combining an individual desire for change with deliberate action where individuals attempt to accomplish “external change to relationships, situations, power dynamics, or contexts”. As the process is based on changes that are seen as meaningful by the empowered individual, the facilitation of empowerment requires an understanding of what the individual or community sees as meaningful and why. This understanding is built into the definition of peer support models as the provider and receiver of support share experiences of the problem at hand.

### The Philani model

The Philani Maternal, Child Health and Nutrition Trust is a South African non-governmental organization operating in socially disadvantaged townships in the Western and Eastern Cape provinces. Since 2002, the organization promotes family health through the Mentor Mother Program, which uses home-visits delivered by paraprofessional peer supporters to support and empower pregnant women, mothers and young children. These peer supporters are women who are recruited from the areas where Philani operates, who themselves have been able to raise healthy children despite challenging circumstances.

The peer supporters are trained in basic maternal and child health and behavior change techniques. Their clientele is recruited by visiting the homes of all mothers in geographically defined areas. During home visits they work as generalists to support a number of health behaviors relating to child nutrition and development, HIV prevention, alcohol misuse and mental health, and refer family members to clinical services as needed. They also assist in securing child financing assistance grants.

The model’s philosophy is summarized in five key pillars [19]:

**•** A careful recruitment process: The program identifies women who can act as role models in the community, using a careful internal interview process to select suitable candidates.
**•** Appropriate training: The recruited women receive an initial training of six weeks, recurring training in the field and monthly meetings with a training component. The Department of Health provides parts of the training.
**•** Home-based, action-oriented health intervention: Rather than solving their clients’ problems, the peer supporter shares her knowledge and coping skills to help families’ find their own solutions to problems they are facing.
**•** In-the-field supervision and support: Each peer supporter regularly receives in-the-field supervision and feedback on performance. Time is also set aside for debriefing particularly difficult cases with coordinators.
**•** Monitoring and performance feedback: Outcomes such as rehabilitation rates, exclusive breastfeeding rates and antenatal clinic attendance are monitored closely and used to measure the effectiveness of the intervention.

Program evaluations have found the intervention to be effective in improving a number of maternal and child health outcomes [20, 21]. Since 2012, the program has been adapted and extended to Ethiopia, Eswatini and Egypt. During 2021, the model was implemented in Sweden, where the implementing organization Yalla Trappan adapted the model to fit the local context. This process of contextualizing the model involved joint definitions of the target group, overall project objectives, practical activities, and strategies for community outreach. The transfer of the model to the Swedish setting was initiated by the Church of Sweden in 2018. Their extensive involvement with socially vulnerable groups in Sweden and experience of supporting Philani's work had indicated that there was a demand for both individual support for health issues and for empowerment through social inclusion in the Swedish context, and that the Philani model could potentially meet these needs.

### Aim

Transferring social innovations from low- and middle- to high-income countries challenges norms around producing and implementing knowledge and new practices. Nevertheless, the same conditions for scaling up – push and pull factors, strategies to connect the two, and contextual adaptations – are required when social innovations are being transferred to new settings [13]. Understanding how this transfer happens may open up for new knowledge exchanges and practices dissemination in global health. This study aimed to document and analyze the process of adapting the Philani model to the Swedish context.

## Methods

### Study context and participants

This study was conducted as a part of an ongoing project centering on the implementation of the Philani model in Malmö, southern Sweden. The project’s steering committee involved stakeholders from four organizations within different sectors:

• Yalla Trappan (YT), a social enterprise creating opportunities for integration on the labor market for women within migrant communities.
• The Preschool Department in Malmö city (PD), involved in several initiatives within early childhood education and social sustainability.
• The Church of Sweden (CoS), whose stakeholders had a long experience of supporting Philani’s work in South Africa.
• The Department of Women’s and Children’s Health at Uppsala University (UU) represented by the first (PK) and last author (MM), the latter being the previous manager of Siphilile Maternal and Child Health, a non-governmental organization in Eswatini implementing an adapted version of the Philani model since 2012. The authors' role in the committee was to provide knowledge support and facilitate a structured implementation process.

The project builds upon a similar initiative that was implemented during 2019–2020 by PD and YT, involving the same steering committee and employing the same peer supporters, focusing on guiding disadvantaged families to open preschools through community outreach. These preschools offer a free platform for families to participate in pedagogical activities and language training for both children aged 0–5 years and their parents. Since January 2021, five peer supporters are employed by YT, who are responsible for the implementation of the current form of the project. The recruited peer supporters have different cultural and linguistic competencies to match the populations in socially disadvantaged areas in the city. The peer supporters have weekly group meetings with a coordinator, which offers an opportunity for debriefing and supervision.

Malmö is Sweden’s third largest city. The population is multicultural, with 46% of inhabitants being born abroad or having two parents being born abroad [22]. The city is also socially and residentially segregated [23]. A previous study in the region demonstrated large socioeconomic differences in unmet health care needs, aggravated by lack of trust in the health care system, lack of interpersonal trust and economic stress [24]. The health consequences of the segregation have also been thoroughly assessed by the Commission for a Socially Sustainable Malmö, publishing a report in 2013 displaying how a number of child and adult health outcomes varied greatly between different city districts, following a distinct social gradient [23].

### Data collection

Qualitative data were gathered from workshop sessions, providing data on the contextualization process, and previously held interviews, covering challenges and opportunities for community outreach in Malmö. The use of two sources of data allowed for multiple perspectives.

Three online workshops were held in February and March 2021, each session lasting 110–210 min. All project steering committee members from YT (*n* = 3), PD (*n* = 3) and CoS (*n* = 2) were included as participants. The workshops focused on how to adapt the Philani model to the Swedish setting, by formulating a logical framework specifying inputs (resources and training), outputs (activities), outcomes (desired results) and an impact (overarching vision). All participants had previous knowledge of the Philani model, which was enhanced during a meeting with representatives from Philani, taking place between the first and second workshop. Workshops were used to generate qualitative data and allowed for a variety of described experiences, views and opinions, where participants could establish shared positions and collective understandings of problems through intersubjective interaction [25]. The workshop process thereby aimed to generate stakeholder consensus and provided a data collection opportunity. All sessions were audio recorded. Observer notes were taken continuously during the workshops, noting down preliminary ideas and themes.

Eight semi-structured interviews were conducted online in June and July 2020 as a part of documenting experiences of community outreach in Malmö. Interview questions centered on how hard-to-reach families were approached and engaged, and barriers and facilitators for promoting preschool attendance and social integration at large. The interview guides are available in Additional file 1. The interviewees included all people working with the peer support project in Malmö at the time, namely peer supporters (*n* = 3), stakeholders from YT (*n* = 3), and stakeholders from PD (*n* = 2). The interviews lasted 23–79 min and were audio recorded.

All participants in the workshops and interviews were women, as no men were employed as peer supporters or participated in the steering committee, with the exception of the researchers. All recorded material was transcribed verbatim.

### Data analysis

The data were analyzed using thematic analysis, following the six steps outlined by Braun and Clarke [26]. Thematic analysis was considered appropriate as the data from the workshops were not structured according to pre-set questions, and the interviews provided data that in part contained material outside the scope of this study's aim.

The interview and workshop transcripts were coded inductively using Microsoft Word, generating descriptive codes. A subset of coded material was discussed between the authors to ensure validity. Codes were grouped and condensed inductively into themes and subthemes, repeatedly going back to the coded data to ensure their validity. The themes were reviewed and revised during three meetings with the authors. Examples of how codes, sub-themes and themes were generated from the data are available in Additional file 2.

### Methodological considerations

Using workshops as a source for qualitative data allowed for data collection in a negotiated process, where shared and personal views were clarified and discussed with the aim of developing stakeholder consensus. The qualitative data gathered during the workshops were by nature unstructured and scattered, as they were not based on pre-formulated interview questions. This presents a limitation during thematic analysis, as the data did not offer the possibilities of depth that interviews or focus groups can do.

The workshops did not include the peer supporters themselves, due to stakeholders' preference to focus on overall vision and organizational goals rather than operational aspects. This is a limitation as their understanding of the context offers a depth that is difficult to achieve otherwise. Their perspectives were however captured during the interviews.

Interviews were not conducted with the recipients of the intervention, as the focus of the study was to understand how the Philani model was adapted by stakeholders in the implementing organization.

The interviews were held by PK, who is a male medical doctor from Sweden. Although he had met and come to know all the interview participants on previous occasions, it is possible that the power dynamics of the interview situation influenced what the participants, particularly the peer supporters, were comfortable expressing. However, during the interviews, all participants were able to problematize and discuss difficulties in detail. The workshops were attended by PK and facilitated by MM. MM is also a male medical doctor from Sweden, and a professor of global health. He also has previous experience of working with implementing the Philani model in Eswatini in Sub-Saharan Africa. Ideally, workshops provide a platform for free discussion and innovative ideas, but different levels of previous experience can naturally lead to difficulties in speaking up or questioning what is being discussed. Compared to the first workshop, participants had a more even distribution of time spent speaking during the second and third workshops, suggesting that this effect diminished over time.

### Ethical considerations

The Swedish Ethical Review Authority approved of the study (2020–04288). Interviewees and workshop participants were provided written and oral information and were given chances to ask questions about the study. Participants were also informed that they could withdraw participation at any stage without having to state a reason and without consequence. All participants signed an informed consent form. The transcripts were anonymized and stored on protected university servers, available only to the researchers. The participants did not receive financial compensation for their participation.

Some workshop participants had previous experience of working with the Philani model while others did not. Similarly, during interviews there was a discrepancy in theoretical knowledge of the Philani model between interviewer and interviewees. To prevent this from influencing the thoughts and opinions that participants were comfortable expressing, their own expertise in the local context was emphasized.

## Results

Five themes were generated in the thematic analysis, representing different aspects of the contextualization process:

- (1) *Focus on the social determinants of health*, describing how the intervention content and the definition of the target group was centered on underlying socioeconomic factors rather than health behaviors,
- (2) *An organic systemic approach*, in which the intervention logic built on indirect mechanisms and social ripple effects to achieve change,
- (3) *Linking to existing services*, in which the working methods built on referrals to established social and health services,
- (4) *Matching peer supporters with the community*, which considers how the heterogeneity of the sociocultural context required diverse language and cultural competencies among the recruited peer supporters in order to build trust, and
- (5) *An intervention governed by flexibility*, where a broad and flexible approach to tackling social challenges let the peer supporters and supported women themselves determine their needs and how to meet them.

**Fig. 1:**
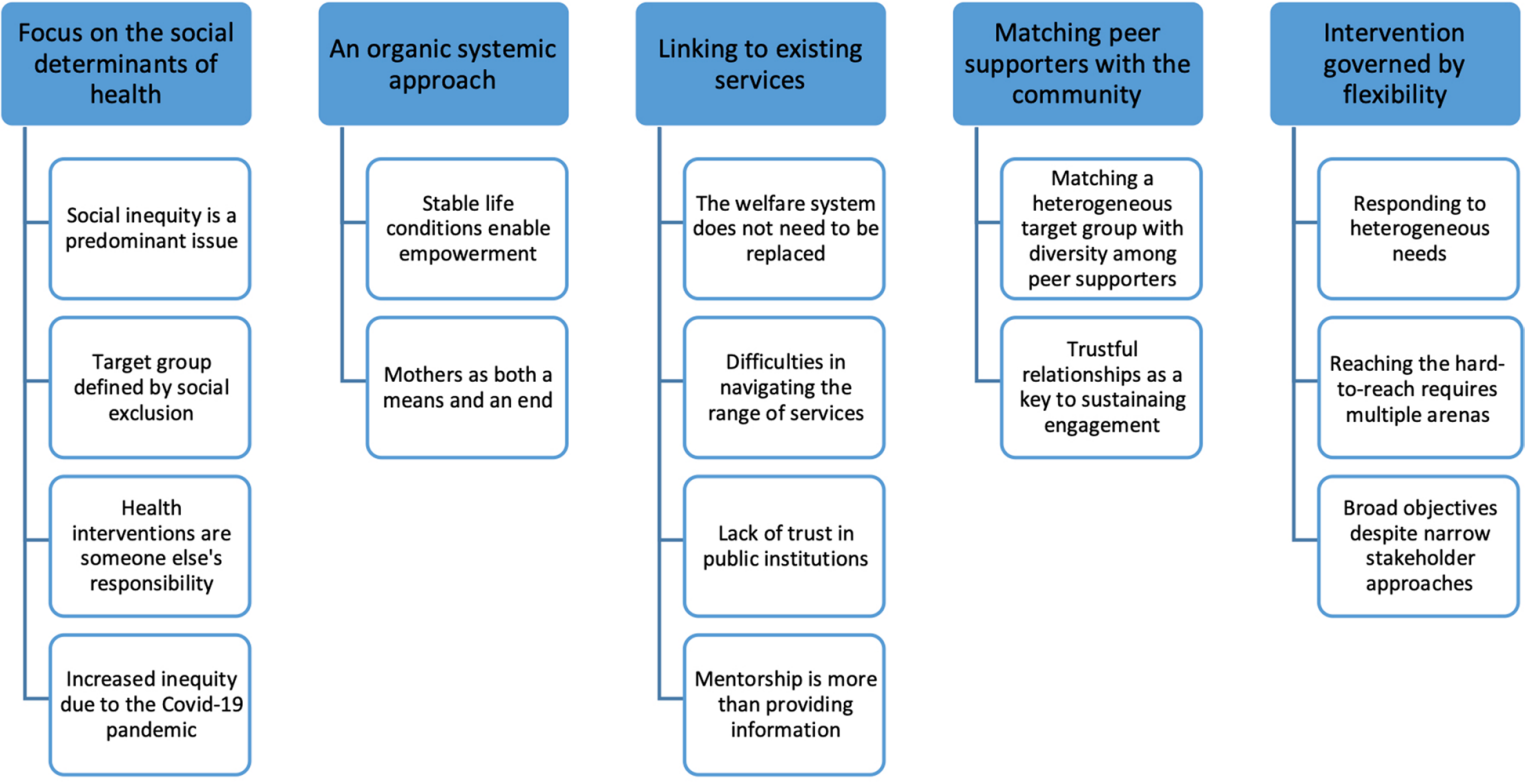
Themes and sub-themes

Fifteen sub-themes were discerned within the five themes, outlining specific elements of the same organizing concepts. These sub-themes described perceived contextual factors, rationalizations, and personal experiences of working in the environment. The themes and corresponding sub-themes are presented in Fig. 1.

### Theme 1: focus on the social determinants of health

The first theme described how participants highlighted the importance of improving social determinants of health in the target community in Sweden and how this was contrasted with direct interventions for health.

Social disadvantage in terms of living conditions, education, income and participation in the Swedish majority society were put forth as pressing issues to prioritize to increase quality of life in the area where the peer supporters were working. This justified an intervention focusing on these factors, with indirect effects on family well-being and health, rather than health behaviors directly. Participants expressed that those most in need of parental support in the Swedish environment were those most affected by social marginalization. It was agreed that the most effective target group to focus on would be mothers of young children and pregnant women with an immigrant background who needed support to access the various elements of the welfare system and increase their participation in Swedish society.

*“It’s this group that’s really the purpose then. They are the furthest from society, most difficult to reach for all kinds of social institutions and with information about society, and yes, with everything that has come up now with vaccinations and [Covid-19] testing and everything like that, this is the group that is constantly found to be the furthest away from society.” [Workshop 2:9]*

Drawing on participants' previous experience of working in the area, the lack of social platforms outside the home among families in the project's target group was raised as an important factor for their overall well-being, contributing to the perpetuation of social and health inequalities. Focusing on improvements in social determinants of maternal and child health rather than direct interventions for health was further motivated by the existing range of services within the health sector, and by the participants’ own professional backgrounds.

While both health and its social determinants such as social capital and employment were described as evidently inequitable in this context, participants also described how the Covid-19 pandemic had exacerbated such societal gaps through increased social isolation and higher thresholds for accessing the range of available support services.

### Theme 2: an organic systemic approach

The second theme concerned the intervention logic, where improvements in the social conditions of mothers and children in the target group were discussed in terms of ripple effects, with impacts extending beyond what the peer supporters' activities directly addressed.

Participants further expressed that in order to overcome social inequalities using the Philani model, with its intervention logic emphasizing individual agency rather than changing societal structures, empowerment was a key concept. In order to empower individuals to take charge of their own lives, it was considered essential to help them overcome constraints such as financial difficulties and limiting or abusive relationships.

Participants described how peer support for parents can lead to improvements in both the parents' and the children's social environment. Empowering parents was discussed both as an end in itself and as a way to improve children's well-being.

*“So, the goals are for the parents, but the parents are also a means to an end. They are the means for the children to be empowered.” [Workshop 3:8]*

While the role of fathers in caregiving was described as important, norms surrounding responsibilities within families were also discussed as a factor to consider. Such norms made it natural to focus the model on support for mothers.

### Theme 3: linking to existing services

The third theme concerned how to relate to existing welfare services, and the role of peer supporters in linking families to these.

As the Swedish context offers a range of reliable and accessible services for mothers and children – such as social services, health care, early childhood education and language courses – the role of the peer supporters was adapted to complement these services rather than to replace them. Shaping their professional role in a way that could add value without overlapping with the role of other professionals was an ongoing process, constantly being shaped by experiences of interacting with social workers and open pre-school pedagogues.

Participants voiced that the lack of easily accessible information on available public services was a barrier for the target group to identify opportunities for social inclusion. This resulted in difficulties in accessing maternal and child health care, pre-school, social services, the education system and the labor market. One aspect of these difficulties in navigating Swedish society was language barriers. Participants further described low trust in public institutions as a contributing factor to the low utilization of public support systems by the target group. This was seen as a barrier to linking the target group to available services, with implications for social inclusion in general.

Providing information about where families could get different types of support was highlighted as one aspect of the model, but an even greater value that the Philani model could offer in the Swedish setting was described as contributing to a sense of belonging and security. An important aspect of this was that peer supporters could lower the threshold for using welfare services and educational programs by physically accompanying them to appointments and activities.

*“To dare to take the step towards what you don't know, to be confident in that, and that the peer supporter can be a support in that effort. And perhaps apply for Swedish for Immigrants [language course], find out what support is available from the employment agency and get in touch with various support services such as women's shelters. [...] Being a support all the way.” [Workshop 3:2]*

### Theme 4: matching peer supporters with the community

The fourth theme focused on how social, cultural and linguistic heterogeneity in disadvantaged areas required diversity in recruitment, in order to respond to the needs of the target group with appropriate competencies and to facilitate the building of trusting relationships.

Matching peer supporters with supported families based on language, shared understandings of cultural norms and values, and shared experiences of migration were described as keys to successful support.

*“A lot of it is about language and culture, that you have a common understanding, you come from the same culture, you share the same background in some way. You support each other, and it gives a lot of families a sense of security to be able to identify with another woman who has the same background, the same language and the same experiences.” [Interview 7]*

Participants underscored how this matching enabled the peer supporters to gain a deeper understanding of the needs of the supported mothers. Along with both personal qualities of the peer supporters and an emphasis on confidentiality in meetings with the target group, this matching enabled them to build trusting relationships, which were described as important factors in initiating contact and maintaining engagement among families.

### Theme 5: an intervention governed by flexibility

The fifth and final theme centered on how the adapted Philani model had a broad objective and a high degree of flexibility at all levels, allowing for individual tailoring of intervention content and working methods.

The social challenges highlighted by the participants – e.g. social isolation, language barriers to accessing health services or lack of knowledge of the welfare system – were described as very individual, and the Philani model was considered as an innovation that could be implemented with considerable flexibility regarding how to address them. This flexibility was described in regard to what type of support was provided to individual families, thereby adapting to the needs of the target group rather than following a fixed agenda.

Flexibility was also discussed with regard to working methods in the field. As the target group was defined in terms of social exclusion, the need to use several arenas as contact points to reach them was stressed. These venues included welfare and education services such as open preschools, child health care services and social services. They also included informal networks and public places, which the peer supporters had previously used successfully to establish contact with new families. The use of informal networks relied on the peer supporters' own knowledge of the social context in which they operated.

*“One mother knows the function of the peer supporters and yes, but my friend, she needs [support] too. So, it's within these unofficial networks that the most things are happening, I would probably say. It’s like there you actually get direct contact.” [Workshop 3:9]*

Although the stakeholders’ individual domains of interest and expertise were delimited and focused, they were considered to fit well into an intervention with a broad objective that could accommodate many different components and types of activities. This broad and flexible approach to addressing social challenges allowed the peer supporters to respond to many different types of needs and adapt their strategies as they saw fit.

## Discussion

The findings of this study point to a multifaceted range of considerations made when contextualizing a community-based peer support model for disadvantaged families from a low-income country to a high-income country. These considerations resulted in adaptations on several levels: the overall goal was formulated to focus mainly on social factors rather than health outcomes, the procedures for achieving this goal were based on linkage to services rather than behavioral change, peer supporters with heterogeneous competencies were recruited, and a high degree of flexibility was adopted regarding both the location, frequency and duration of meetings. The findings illustrate how the internal and external context – e.g. the conditions within the organization implementing the model and in the environment to which the model is adapted – influence what is possible and appropriate to do within the model’s framework.

### Recurring priorities in high-income contexts

The themes outlined in our results reflect the strategies of some similar programs in high-income countries. These include linking families to other services and improving social determinants such as financial stability, which are components of home visiting interventions such as the Early Head Start and Healthy Families America programs in the US [27]. The latter has also seen a higher retention rate when matching clients and providers based on ethnicity [28], reflecting the priorities highlighted in our findings. Furthermore, flexible field methods allowing for individual tailoring of content, sometimes described as ‘precision home visiting’ [29], are used by interventions such as Family Spirit, a program that serves disadvantaged Native American families [30]. Previous reviews have also highlighted how several paraprofessional home visiting programs have used individual tailoring of the frequency of meetings, mode of contact and location [17], and how the need for such flexibility is greater among highly vulnerable families [31].

### Adapting support programs to new settings or groups

The results of our study describe how the Philani model was discussed, received and adapted without a systematic approach to its core components. Our analysis thus provides insight into the results of the informal adaptations to the context in southern Sweden, rather than offering descriptions of how deliberate changes were made based on considerations of differences in sociocultural and material context between Sweden and South Africa.

Previous studies on contextualizing paraprofessional support for families have often focused on external context in the form of cultural aspects at the provider level, to ensure that the content is relevant and delivery takes place in an appropriate and effective manner [32–34]. Our results highlight how adaptations can also be made in relation to the wider social context, such as the availability of welfare services, which is less commonly described. This landscape of public services can influence both ‘pull’ and ‘push’ aspects of social innovation transfer, as described by Mulgan and Pulford [13]. The availability and use of services influences the demand for new social innovations, perhaps in particular for third sector initiatives, as these can compensate for the rigidity of public welfare [14], e.g. through a focus on linking clients to these services. Availability of platforms for cross-sectoral cooperation – such as open preschools in this case – also influence the feasibility of implementing social innovations focused on community outreach. The social context in the form of vulnerable groups with heterogeneous needs further influences the transfer process, where flexible approaches to meeting recipients’ needs could be seen as a strategy for connecting demand and supply in social innovation.

Few previous studies have explored the process itself in informal adaptation of paraprofessional support, and previous research has tended to focus on how informally adapted interventions are received [32] or how interventions can be systematically adapted through involvement of community members or stakeholders [30, 33, 35]. Our process differed from these mainly due to the fact that the ownership of the intervention, and thus its contextualization, resided with a different organization than the research project. Understanding the process of adapting social innovations outside the formalized structures of academia can shed light on the role of the third sector in cross-contextual transfer of knowledge and practices. This knowledge is important as a large portion of social innovations are implemented by actors such as non-governmental organizations [36].

### Comparison to Philani’s five pillars

While adaptations of parental support interventions can allow for reaching new groups and increase retention, the positive effects cannot be assumed to remain if core elements are changed or replaced [37]. This is a potential risk when interventions are adapted informally, and it is important to clarify these before changing the intervention’s content or how it is delivered. While not articulated as the core of the model per se, the five pillars of the Philani model describe its fundamental operational priorities [19]. To understand the extent to which the model was adapted, our results can be interpreted in relation to these.

The first pillar is a careful recruitment process. This includes recruitment of women who can act as role models in the community, as well as a careful selection after an initial evaluation. A sub-theme in our analysis was *trustful relationships as a key to sustaining engagement*, where participants expressed how the ability to instill a sense of trust was fundamental to achieve good peer support, and a personal quality to consider when recruiting. The ability to build trust can be seen as one way of acting as a role model, even if it does not regard motherhood per se. The sub-theme *matching a heterogeneous target group with diversity among peer supporters* described how responding to heterogeneity in the Swedish setting was a prerequisite for building this trust. This matching necessitated an adaptation of how recruitment was undertaken, without challenging the careful selection process itself. The possibility of selecting potential peer supporters after initial internal evaluation was not discussed during the workshops or interviews. The need to match high cultural and linguistic diversity may however limit the possibility of choosing a large number of candidates. Strong legal protection in Sweden also prevents dismissal of candidates shortly after employment [38].

The second pillar is appropriate training. In the South African setting this consists of six weeks of training, preparing for the range of tasks that the peer supporter may perform in the role of a health and social worker. To ensure that the training received by the peer supporters is appropriate, it must naturally correspond to the tasks and challenges they are likely to face in the field. The South African setting presents challenges such as a high burden of HIV [39] and undernutrition [40]. In the Swedish context, the prominent challenges were described as social isolation, marginalization, unemployment and barriers to accessing social services, requiring a *focus on the social determinants of health*. This mirrors how bridging social gaps in child health has previously been framed in urban areas in Sweden, such as by the Commission for a Socially Sustainable Malmö [23]. Meeting social needs by linking to the right services requires a good knowledge of the structure of Swedish society and what types of support and opportunities are available within both public and civic sectors, which are important aspects to consider in training. Peer supporters must also have an understanding of why e.g. participation in early childhood education makes a difference to children's development, if they are to empower families in their decision-making. The training provided within the adapted Philani model was structured to cover these aspects.

The third pillar is home-based, action-orientated health intervention, which represents both what is done during meetings, how it is done and where. Home-visiting may be a viable strategy to recruit clients and maintain their engagement in a setting where they cluster together geographically, as is the case where the Philani model is developed [41]. The sub-theme *target group defined by social exclusion* in our analysis described how recipients of the model in Sweden were defined based mainly on individual characteristics rather than primarily on the basis of living in a disadvantaged geographical area. This has implications for how these families are reached in the Swedish context, as addressed within the sub-theme *reaching the hard-to-reach through multiple arenas*. When peer supporters cannot rely on spatial delimitations, their strategies for initiating contact with potential clients must be multi-faceted and rely on their own understanding of the social environments of the target group. This represents a shift in the level on which decisions are made, as peer supporters cannot be assigned a well-defined geographical area by a supervisor.

Changing the focus of the model from promoting health behaviors within the family, as in South Africa, to a strong focus on *linking to existing services*, as described in Sweden, marked a further shift away from a home-based intervention, demonstrated by the emphasis on Swedish peer supporters physically accompanying mothers to appointments and activities. A factor not discussed during workshops or interviews was how individualistic norms may affect the use of the home as an arena for interventions. Sweden has traditionally been described as a deeply individualistic society despite a strong emphasis on collective solutions [42], where individualistic values are particularly strong in the private sphere [43]. This could have a negative impact on the possibility of contacting families by seeking them out in their homes.

The choice to prioritize improvements in social conditions over direct health intervention was described as necessitated by the outer context in Sweden – both the needs of the community and the widespread availability of preventive and curative health services, where the latter were something the peer supporters had to complement rather than compete with, by prioritizing linking. This ability to complement public systems, sometimes compensating for their inability to meet diverse needs and minority preferences, has been highlighted in previous research on the role of the third sector in welfare [14]. The internal context of the organization, in terms of stakeholders' professional expertise, which was principally in integration, social sustainability and education issues, also influenced the decision to prioritize social determinants. This very central change in what the intervention was formulated to achieve thus partly emanated from the inner context of the implementing organization itself and may not be fully applicable to other actors in Sweden.

The fourth pillar is in-the-field supervision and support. Supervision was not addressed directly by workshop participants and only mentioned in passing during interviews. However, the sub-theme *matching a heterogeneous target group with diversity among peer supporters* in our analysis identifies considerations to take into account. The need for diversity in language skills and cultural competencies of the peer supporters require corresponding competences of the person providing supervision in the field if they are to have a comprehensive understanding of how meetings with clients are conducted. This may complicate adherence to the fourth pillar when it is adapted to fit a multicultural setting. Accordingly, peer supporters in the adapted intervention are not supervised in the field but instead have weekly group meetings with a project coordinator.

The fifth pillar is monitoring and performance feedback. Measuring standardized clinical outcomes such as breastfeeding rates or HIV treatment fidelity in the target population allows for straightforward evaluation of the effectiveness of the intervention at the recipient and provider level. The data collected within this study mainly concerned strategic considerations, and while monitoring in the Swedish setting was not described directly, some considerations touch upon potential challenges. An intervention aimed at improving social conditions in a broad sense may require a range of outcome measures to give a representative picture of what has been achieved. Another potential barrier to systematic monitoring is flexibility on the level of the provider-recipient interaction. The more flexible an intervention is in relation to the number of problems it is intended to address, the more difficult it may be to monitor and quantitatively evaluate its success [44]. The sub-theme *responding to heterogeneous needs* points towards this challenge, as participants agreed on the importance of peer supporters tailoring their work to individual needs. In the adapted intervention, the monitoring takes place through registration of digital forms. These include both pre-set response options and free-text fields for reporting field activities, with the pre-set options being continuously reviewed and updated to reflect the activities being reported.

Our analysis of the contextualization process thus suggests adaptations of the five pillars of the Philani model. While core elements such as careful recruitment, appropriate training and supervision were contextualized while still remaining relevant, the intervention content centered on a home-based health intervention was modified to a large extent to respond to needs in the context at hand. In line with its philosophy in its original context, the model implemented in Sweden did however emphasize empowerment of pregnant women and mothers of young children as a key to overcoming barriers to their own and their children’s health and wellbeing, by building on strengths within the community, harnessing peer supporters understanding of the local context and their target group and using trustful relationships to engage and retain supported mothers.

## Conclusion

The South African Philani model was contextualized to support mothers of young children and pregnant women in socially disadvantaged immigrant communities in Malmö in southern Sweden. During the process, adjustments were made based on various contextual factors. Pressing social needs, such as lack of social integration, motivated a broad focus primarily on social determinants of health rather than on medical issues directly. This broad focus warranted adapting a systemic approach to clients’ needs. The range of available welfare services justified an emphasis on linking clients to these rather than directly intervening to change their health behaviors. The multicultural nature of the target communities required the employment of peer supporters with diverse languages and cultural competencies. The diversity of stakeholders, the definition of target group in terms of social rather than geographic characteristics and diverse needs of the clients justified flexible content and working methods. This study thus highlights the range of considerations that can arise when a social innovation developed in a low- or middle-income context is implemented in a high-income context.

## Supplementary Information

**Additional file 1.** Topic guide – Interviews with stakeholders at Yalla Trappan and the Preschool Department.

**Additional file 2. **Examples of how codes, sub-themes and themes were generated form the data. 

## Data Availability

The datasets generated and analyzed during the current study are not publicly available due to the absence of such an agreement with the study participants, but are available from the corresponding author on reasonable request.
