# Supplementary material for: Adapting a South African social innovation for maternal peer support to migrant communities in Sweden: a qualitative study
Source: Int J Equity Health. 2022 Jun 22;21:88. doi: 10.1186/s12939-022-01687-4 (PMC9217115; doi:10.1186/s12939-022-01687-4)
Supplement: Supplementary file 1 — Additional file 1. Topic guide – Interviews with stakeholders at Yalla Trappan and the Preschool Department. [file 12939_2022_1687_MOESM1_ESM.docx]

## Topic guide – Interviews with stakeholders at Yalla Trappan and the Preschool Department

**Introduction**

- Introducing the interviewer and Uppsala University's research part of the Philani project
- Brief presentation of the study topic – to capture the process of The Early Life and the work of peer supporters to date
- Explaining anonymity and confidentiality
- Explain recording, length of interview (approx. 60 min), interview set-up, output of study and data management
- Review consent to interview participation and their right to discontinue participation or skip questions at any time
- Are there any questions before we begin?
- Check that they are comfortable with proceeding to the interview and recording

**The initiation of The Early Life**

- What prompted the initiative?
  - What needs were you aiming to meet?
  - Were there other initiatives trying to meet the same needs?
- How did the start-up process work?
  - Were there any challenges that you identified before the initiative started?
- How did the cooperation start?
  - What led to Yalla Trappan becoming a partner?

[Questions specifically for Yalla Trappan]

- *How did you go about recruiting peer supporters?*
  - *How was the training of new peer supporters carried out?*
- *Was there anything that was challenging during the start-up of the work with the peer supporters?*

**Ongoing work within the project**

- What does your own work with The Early Life and the peer supporters look like in practice?
  - How do you perceive the project to be functioning today?
    - What part of the work do you feel is most worthwhile?
    - What are the biggest challenges at the moment?
  - Where do you see the greatest unmet needs in the communities you are working with?

**Contact with families**

- How do you feel the work of Early Life is received by the families you work with?
- Do you find that some target groups are easier or more difficult to contact or engage than others?
- How has the work been adapted based on how it has been received by the target group?

[Questions specifically for Yalla Trappan]

- *How are the peer supporters supervised?*
  - *How do you deal with peer supporters who encounter practical difficulties in their work?*
  - *How do you deal with the emotional strain on peer supporters in their work?*

**Vision of the future**

- How has the collaboration with the Church of Sweden and Uppsala University influenced the vision of what you want to achieve with the peer supporters?
- What are your hopes for the collaboration within the project?
- What do you think is required for the cooperation with the Church of Sweden and Uppsala University to be as beneficial as possible?
- What do you think are the largest challenges and opportunities at the moment?

**Finishing the interview**

- Is there anything that you have found important in the process of working in The Early Life that we have not yet addressed?
- Information on what happens now regarding the interview material – transcription, anonymization, data management and output
- Wrap up and thanks for participation

## Topic guide – Interviews with peer supporters

**Introduction**

- Introducing the interviewer and Uppsala University's research part of the Philani project
- Brief presentation of the study topic – to capture the process of The Early Life and the work of peer supporters to date
- Explaining anonymity and confidentiality
- Explain recording, length of interview (approx. 60 min), interview set-up, output of study and data management
- Review consent to interview participation and their right to discontinue participation or skip questions at any time
- Are there any questions before we begin?
- Check that they are comfortable with proceeding to the interview and recording

**Background**

- What is your own professional background?
- How did you get the job as a peer supporter?
- What made you interested in working as a peer supporter?
- What was the training process like?

**Working as a peer supporter**

- What does a typical working day look like?
- What kind of supervision do you receive from Yalla Trappan?
- How have you used your previous personal or professional experience in your work?

**Reaching out to families**

- How do you make contact with a family?
- Is there anything you find challenging in establishing contact?
  - How do you handle that?
- How do you involve fathers in the meetings?

**Working with families**

- What does a meeting with a family look like?
- What do you do to build a relationship with the family?
- How long do you usually stay in contact with each family?
- What aspects of your work do you perceive as most valuable to families?

**Dealing with adversity**

- What do you find most challenging in your work?
  - What do you do when you don't know how to proceed with a family?
  - How do you deal with families who you think might need contact with social services or other authorities?
  - What do you do when a meeting with a family is emotionally distressing?
- What kind of support and supervision do you receive from Yalla Trappan?

**Vision of the future**

- How would you like the project to develop in the future?
  - What do you think is needed to get there?

**Finishing the interview**

- Is there anything that you have found important in the work as a peer supporter that we have not yet addressed?
- Information on what happens now regarding the interview material – transcription, anonymisation, data management and output
- Wrap up and thanks for participation
