# Supplementary material for: Adapting a South African social innovation for maternal peer support to migrant communities in Sweden: a qualitative study
Source: Int J Equity Health. 2022 Jun 22;21:88. doi: 10.1186/s12939-022-01687-4 (PMC9217115; doi:10.1186/s12939-022-01687-4)
Supplement: Supplementary file 2 — Additional file 2. Examples of how codes, sub-themes and themes were generated form the data. [file 12939_2022_1687_MOESM2_ESM.docx]

| **Data extract (example)** | **Code (example)** | **Sub-theme** | **Theme** |
| --- | --- | --- | --- |
| *Then health, that's probably really important too and I... I understand [name]'s views there and so. But it's clear that it's implicit in this, to be able to go to preschool and open preschool and so on. Well, I think it's more omnipotent, or how to say it, more comprehensive to say in that case that this is about creating trust among these women and families that will benefit their children.* | Health inequalities are not the most pressing problem | Social inequity is a predominant issue | Focus on the social determinants of health |
| *…the results in Malmö's schools were so incredibly skewed, I mean, such a huge difference. We had schools that had a secondary school entrance qualification rate of 27 percent and we had those that had upwards of 95 percent. So a tremendous inequality.* | Unequal life conditions in Malmö |  |  |
| *It’s this group that’s really the purpose then. They are the furthest from society, most difficult to reach for all kinds of social institutions and with information about society, and yes, with everything that has come up now with vaccinations and testing and everything like that, this is the group that is constantly found to be the furthest away from society.* | Target group is hard to reach through social initiatives | Target group defined by social exclusion |  |
| *This arena, family centers and open preschools, it is also a safe environment to be in. If you look at the pandemic effects and this, where it is talked about this very thing, that the vulnerable get sick because they have no arenas outside the home, and they do not have contact with health-promoting and safe environments.* | Social platforms outside the home is a prerequisite for wellbeing and integration |  |  |
| *I think our professions are too far from the health issue, I think there are too many pieces missing then.* | Health is important but others are better suited to work with it | Health interventions are someone else's responsibility |  |
| *The pandemic effects and this, it’s talked about this very thing, that the vulnerable are harmed because they don’t have any arenas outside the home, and they don’t have contact with health-promoting and safe environments.* | Increased isolation during the pandemic | Increased inequity due to the Covid-19 pandemic |  |
| *And that is also an obstacle to integration. We have seen that individuals in vulnerable areas test themselves to a much lower degree. There is a much more widespread skepticism about vaccinations.* | Unequal impact of the pandemic |  |  |
| *I also want us to include economic autonomy somewhere. Because that is also a prerequisite for empowerment.* | Economic security is important for empowerment | Stable life conditions enable empowerment | An organic systemic approach |
| *An important ingredient in this project is to create a sense of security for the families we meet to take more steps. By visiting open preschools with these families, for example, you create a new sense of security, a greater sense of security. There is more to that security that opens up new opportunities.* | A sense of security enables integration |  |  |
| *And we've talked about it as the children being at the center of this, but also the mothers as a target group. So the goals are for the parents, but the parents are also the means to an end. They are the means for the children to be empowered. [...] So it's both. And at the same time, if there's a woman who is subjected to violence in the home, she needs to be safe in order to create security for her child.* | Target group defined as mothers and children with foreign background | Mothers as both a means and an end |  |
| *You see only mothers who have the children, only mothers who take the children to the open preschools.* | Gender norms give mothers responsibility for children |  |  |
| *If the parents receive support, the children usually feel better too. But there are many other actors working with that, for example social workers and social services and so on, so we should not go in and compete with them and we should not go in and do their job, but we should be a complement. It's quite important to find that role.* | The role of peer supporters is not to act as social workers | The welfare system does not need to be replaced | Linking to existing services |
| *I say it like this, that it’s not the mission of the peer supporters to improve learning and development for children, that is to say that it is their mission in itself to do that as an educator or so, but the peer supporters are peer supporters who guide and also build bridges within [the organization].* | The role of peer supporters is not to act as pedagogues |  |  |
| *If you have information about society and what rights you have, what help you can get in society, then that is also integration. So I think it's both language and this, what should we call it? The knowledge base about the functions of society.* | Lack of knowledge about what society can offer is a barrier to integration | Difficulties in navigating the range of services |  |
| *And we also work a lot with the language, because we see that as a major obstacle to increased participation, because if you don't know Swedish, it's difficult to take your place and take part in what's available.* | Lack of language skills is a barrier to social participation |  |  |
| *There are some who have these thoughts about the open preschools, that the social services are there, and that the social services will look at how you react with your child and they will take the children away.* | Lack of trust in society and authorities | Lack of trust in public institutions |  |
| *An important ingredient in this project is to create a sense of security for the families that we meet to take more steps as well. By visiting open preschools with these families, for example, you create a new sense of security, a greater sense of security. There is more to that security that opens up new opportunities.* | Accompanying clients is an important component | Mentorship is more than providing information |  |
| *It may also be that [families] need help to make an appointment at the health center, that sort of thing also comes up a lot. Or at the dentist or something.* | Providing support in contact with health care |  |  |
| *To have an okay Swedish, so that you could be a mediator from Swedish to another language. And then you would also belong to one of the major languages used in Malmö, and that is Arabic, Somali, Dari, Pashto perhaps, and... So the main languages.* | Language skills are important in recruitment | Matching a heterogeneous target group with diversity among peer supporters | Matching peer supporters with the community |
| *The peer supporters also dress in the same way. Well, even the dress code is right in this case. Now it's Ramadan, for example. That's the first thing you open with, so the first topic of conversation. How has it been now during Ramadan? How is it going for you? How is the fasting going? The peer supporter handles that.* | Match peer supporters with the target group is key to success |  |  |
| *Being a peer supporter, it’s… It’s not just to help, but to give them love, to give them everything. To have good contact with them, good behavior with them, to listen to them. Even if you can’t give advice, just listening to them is also great.* | Building relationships is key to success | Trustful relationships as a key to sustaining engagement |  |
| *When I meet with families, I usually tell them that what we talk about between me and you, it should stay here. It will stay here, I won't tell anyone else. I'm not going to tell the community. Maybe I'll tell my boss, to report and so on. But there's no [registration of your] name and no one will know it's you. And that's what builds trust and they feel safe with me.* | Importance of communicating confidentiality |  |  |
| *We do not have to state what a good life is, because what is good for one is not good for another. So, this thing about empowerment and self-actualization and everything, that everyone creates a life that is good for themselves, but you should have the opportunity to do so.* | Heterogeneous needs in the target community | Responding to heterogeneous needs | An intervention governed by flexibility |
| *You can also see where the needs are greatest. And the question is how demand-driven a project like this can be. And it can be quite a lot really, that you see that these are the needs for what we want to achieve.* | The value of a needs-driven intervention |  |  |
| *I think it really should be a breadth of all this that we're talking about. Both open preschools, family centers, social services and playgrounds and parks and shopping centers and everything.* | Using different venues to connect with families | Reaching the hard-to-reach requires multiple arenas |  |
| *One mother knows the function of the peer supporters and yes, but my friend, she needs [support] too. So it's within these unofficial networks that the most things are happening, I would probably say. It’s like there you actually get direct contact.* | Reaching the target group through informal networks |  |  |
| *And I also believe that we can reach a common impact, if you want to call it that. A common vision for it, or a common... Because then we can work, maybe, based on who we are, on the next level, for outcomes or outputs, to kind of bring it together. But we still have the common vision that we carry.* | Possible to find a common goal | Broad objectives despite narrow stakeholder approaches |  |
| *I think for this pilot project that we are in, to create empowerment, doing it on a very broad basis.* | Horizontal rather than vertical intervention |  |  |
